# Open Database for Accurate Upper-Limb Intent Detection Using Electromyography and Reliable Extreme Learning Machines

**DOI:** 10.3390/s19081864

**Published:** 2019-04-18

**Authors:** Vinicius Horn Cene, Mauricio Tosin, Juliano Machado, Alexandre Balbinot

**Affiliations:** Programa de Pós-Graduação em Engenharia Elétrica da Universidade Federal do Rio Grande do Sul, Avenue Osvaldo Aranha 103, Porto Alegre 90035-190, Brazil; mauricio.ctosin@gmail.com (M.T.); julianoc.machado@gmail.com (J.M.); alexandre.balbinot@ufrgs.br (A.B.)

**Keywords:** EMG, feedforward neural networks, extreme learning machines, non-iterative classifier, reliability, prosthetic hand

## Abstract

Surface Electromyography (sEMG) signal processing has a disruptive technology potential to enable a natural human interface with artificial limbs and assistive devices. However, this biosignal real-time control interface still presents several restrictions such as control limitations due to a lack of reliable signal prediction and standards for signal processing among research groups. Our paper aims to present and validate our sEMG database through the signal classification performed by the reliable forms of our Extreme Learning Machines (ELM) classifiers, used to maintain a more consistent signal classification. To perform the signal processing, we explore the use of a stochastic filter based on the Antonyan Vardan Transform (AVT) in combination with two variations of our Reliable classifiers (denoted R-ELM and R-Regularized ELM (RELM), respectively), to derive a reliability metric from the system, which autonomously selects the most reliable samples for the signal classification. To validate and compare our database and classifiers with related papers, we performed the classification of the whole of Databases 1, 2, and 6 (DB1, DB2, and DB6) of the NINAProdatabase. Our database presented consistent results, while the reliable forms of ELM classifiers matched or outperformed related papers, reaching average accuracies higher than 99% for the IEEdatabase, while average accuracies of 75.1%, 79.77%, and 69.83% were achieved for NINAPro DB1, DB2, and DB6, respectively.

## 1. Introduction

Interfaces based on biosignals supported by late developments in areas such as medicine, engineering, computation science, and microelectronics are becoming increasingly popular. Recently, surface Electromyography (sEMG) and Electroencephalography (EEG) signals have been used to offer control of assistive devices for people with some level of impairment, amputation, or specific movement restriction [1,2,3]. Despite the advances in recent sEMG signal classification for the activation of auxiliary devices [3,4,5], optimal signal processing strategies and portable devices development yet face several restrictions. Despite the deterministic range of the sEMG signal in frequency and amplitude, factors such as subject dependency and lack of signal repeatability often preclude efficient and reliable myoelectric pattern recognition and control since the first studies in the area [6,7], making the optimal sEMG signal classification an arduous task from a machine learning perspective [8,9,10,11,12]. Thus, the natural control of assistive devices based on sEMG activation is a field of constant expansion in biomedical engineering.

Usually, open-access sEMG databases have some restrictions concerning the small number of movements performed, the small number of subjects, the lack of assay repetitions, and the variations in the assays themselves. Furthermore, these databases often use different experimental methodologies that make direct comparison among different works unfeasible. The NINAProdatabase [12,13,14,15] was created to compensate some of these limitations by offering data acquired from a large variety of subjects performing different upper-limb movements and, more recently, including repetitions [15]. However, the NINAPro and other sEMG databases frequently offer only the signals processed, but not the routines/algorithms and other methodological tools to help different study groups replicate and create their databases. Thus, we developed a full experimental protocol to offer alternatives to provide not only our database, but also algorithms, code, and experimental material that may help researchers to develop and test their different methods in the use of sEMG for prosthetic control.

In our previous paper [16], a variety of signal representations was explored to establish a more reliable classification procedure for the sEMG signals. This current paper aims to present and validate our sEMG database using the reliable forms of our ELM classifiers used to maintain a more consistent signal classification. Through this work, the first version of our database, which is constantly expanding to include more trials and subjects (including amputees), has been made available to download on our website (https://www.ufrgs.br/ieelab/IEE_sEMG_db.php). Besides the database, we are also making a considerable amount of the complementary material available, such as videos used for the subjects’ stimulation and also LabVIEW routines and MATLAB scripts to enable different groups to recreate the experimental conditions and formulate their databases. Moreover, we hope to help other groups to maintain a relatively close methodology that may make the result comparison more reliable and consistent among the different papers in the area. Currently, our database (denoted IEEdatabase) gathers 48 assays composed of three repetitions of four different trials acquired from four subjects. The different trials explore a different number and order of 17 distinct upper-limb movements repetitions to test the effect of these factors in the signal classification accuracy rate. Additionally, we explore the use of a stochastic filter based on the Antonyan Vardan Transform (AVT) and our concept of reliable sEMG signal classification using two ELM classifiers. The validation of the classifiers is performed using our database and three different NINAPro databases (DB1, DB2, and DB6) for comparison. “Exercise 2” (E2) of the NINAPro DB1 is comparable with our Trial B in movement types, sequential order, and the number of repetitions, while the E2 section of NINAPro DB2 is comparable with our Assay A for the same reasons. For all of our different assays, each subject performed three repetitions, while the DB6 is the only NINAPro database dealing with assay repetitions. On a newer NINAPro database (DB6), a study case involving day and trial repetitions was presented by Palermo et al. [15] focusing on the cross-session (different trials) signal classification, which resulted in considerable accuracy loss, proving the necessity of a regenerative or more reliable classification method to boost the results. Section 2 of this paper details all the methodology describing the IEE database acquisition protocol and the processing techniques. Section 3 presents the results of the validation of the IEE database and all NINAPro databases used through the different signal classification techniques. Section 4 presents the discussion concerning all the results achieved, and Section 5 consists of final considerations and usage notes for the database.

## 2. Materials and Methods

### 2.1. Experimental Protocol

**Figure 1:**
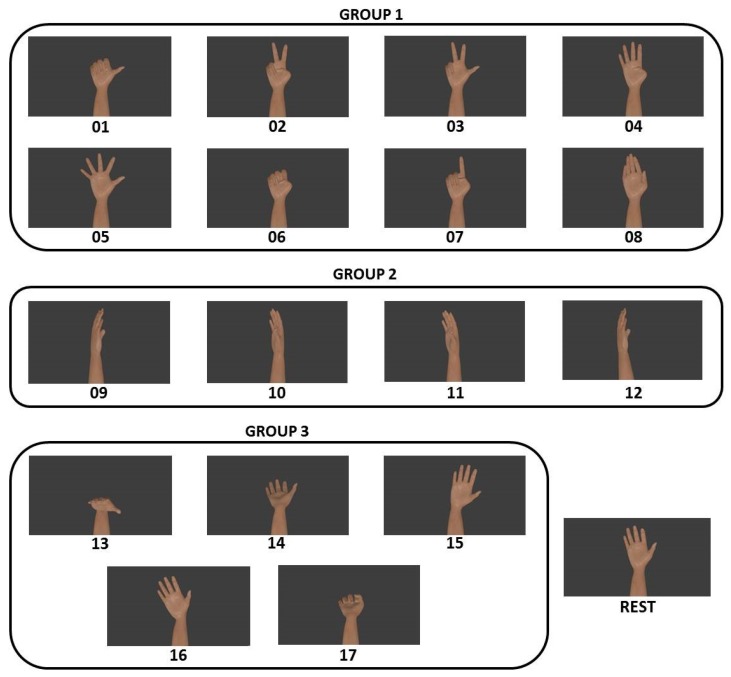
The three different groups of movements performed by each subject in the IEEdatabase.

The E2 NINAPro database exercise [13] inspired the 17 hand and wrist movements that form the IEE database. The 17 different movements performed interspersed by a rest class are presented in Figure 1 and are composed of three main groups. Group 1 consisted of finger movements (thumb up; extension of middle and index finger with flexion of other fingers; flexion of little and ring fingers and extension of the others; thumb flexion; abduction of all fingers; hand closure; pointing index and abduction of extended fingers). Group 2 gathered the torsion movements (wrist supination, axis: middle finger; wrist pronation, axis: middle finger; wrist supination, axis: little finger; and wrist pronation, axis: little finger). Group 3 was composed of wrist movements (wrist flexion; wrist extension; wrist radial deviation; wrist ulnar deviation; and wrist extension with the hand closed).

The characterization of subjects is pertinent to appraise the results since skinnier and younger individuals, for example, tend to reach better accuracy results, as demonstrated by Atzori et al. [14]. During the data acquisition, each one of the four untrained subjects was requested to sit comfortably on a chair positioned in front of an LCD monitor and to reproduce the movements displayed as naturally as possible according to the video stimulation.

**Figure 2:**
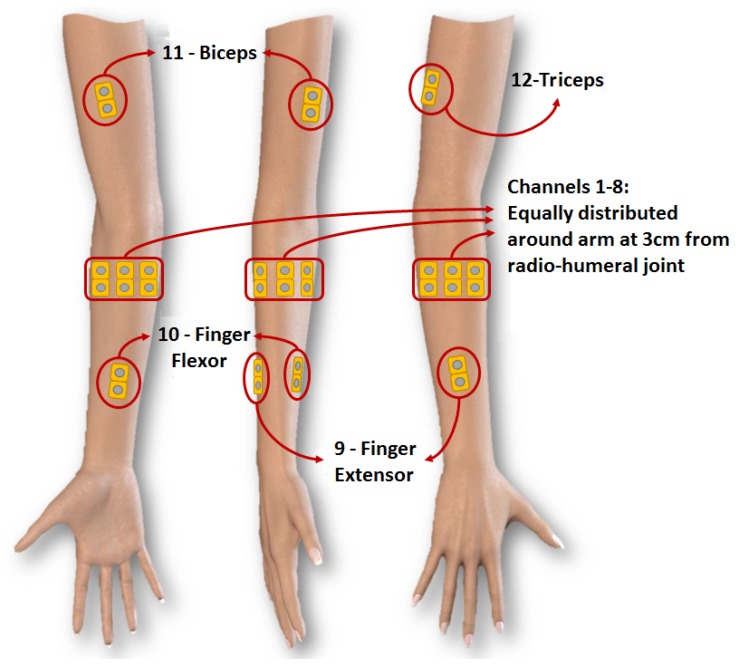
Electrode fixation (Cene et al. [17]).

For each one of the four different assays, the four subjects were requested to repeat the trials three times, forming a subset of the database with 12 trials to each assay. The assays differed from each other according to the number of repetitions and the order of movements. Assays A and B had, respectively, six and ten repetitions of movements in sequential order (as the NINAPro databases DB2 and DB1, respectively). Trials C and D had the same amount of repetitions performed randomly. The electrode positioning was the same as proposed in the NINAPro database, as presented in Figure 2.

All the sEMG signal acquisition was performed by 12 channels formed by 24 disposable surface electrodes and a reference electrode, placed on the individual’s forehead. The channels were connected to a battery-powered commercial sEMG device (*EMG 830 C*, from *EMG System do Brasil*). The signal was digitalized at a 2-kHz sample frequency and 18 bits of quantization by a *NI USB-6289* platform from *National Instruments*. The acquisition was performed using a notebook running *LabVIEW 9* on a *Windows 10* environment.

All procedures performed followed the ethical standards of the 1964 Helsinki declaration and its later amendments or comparable ethical standards and were approved by the institutional research committee under the Certificate of Presentation for Ethical Appreciation (Number 11253312.8.0000.5347). Before the experiment, each subject was requested to give informed consent and answer questions regarding clinical data including age, gender, height, weight, and laterality and also had their Forearm Circumference (F.C) and Forearm Length (F.L) measured. Those characteristics are detailed in Table 1.

### 2.2. The LabVIEW Interface

The LabVIEW routines were designed to interact with the hardware and also provided a cognitive walkthrough to the user, presenting to the user the next movement to be performed in a small auxiliary interface window and providing preparation time. The videos were built using the MakeHuman software for the creation of the anthropomorphic arm forms and the Blender software to put them together and time the animations. The LabVIEW routines and the videos used are available for download on our website along with the MATLAB functions and syntax used to transform the LabVIEW “.lvm” files into “.m” MATLAB files. By making this material available, we hope to enable different groups to recreate the experimental conditions and formulate their own databases, as well.

### 2.3. Data Relabeling

The process of labeling the samples was performed based on the timestamps generated by each movement repetition displayed as the subject stimulation. However, commonly, some delays caused by the volunteer’s reaction time tended to create mismatches and frequently incorrect labels for some samples at the beginning and end of each movement. To improve the signal labeling, an algorithm based on [13], a signal filtering, and a Generalized Likelihood Ratio (GLR) model were used for relabeling.

To define the most proper label in the rest-movement-rest transitions, an exhaustive search between all possible values for the movement beginning and ending time—denoted $t_{0}$ and $t_{1}$, respectively—was performed. The $t_{0}$ was fixed at a determined value based on the initial timestamps, while $t_{1}$ was incremented until the end of the window. After this first iteration, $t_{0}$ was incremented, and $t_{1}$ went through a new sweep. In each iteration, $t_{0}$ was incremented by 30 ms, and the Generalized Likelihood Ratio (GLR) between the rest-movement-rest sections was calculated. Subsequently, the combination of $t_{0}$ and $t_{1}$ was defined for the higher GLR value, which defined the movement beginning and end. The relabeling method, performed in MATLAB, is also available for download on our website.

### 2.4. Signal Filtering

**Figure 3:**
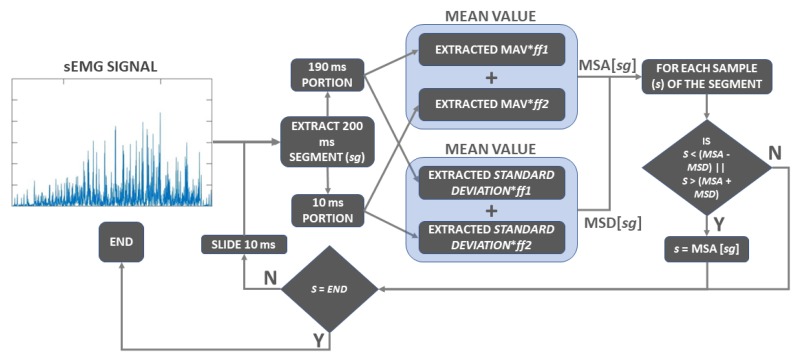
The main block diagram for the Antonyan Vardan Transform (AVT) filter where samples *s* out of the range *Mean Signal Amplitude* (MSA) ± *Mean Signal Deviation* (MSD) are substituted by the MSA value of the signal. sEMG, surface EMG.

Since the noise out of the interesting band of the sEMG signal was usually filtered and removed in the analog conditioning performed in the signal acquisition stage of the process, the digital filtering often aimed to remove artifacts within the bandwidth of the sEMG signal through the application of adaptive filters [18,19,20,21,22,23]. To provide a straightforward and time efficient filter alternative for the sEMG signal filtering, we developed the AVT filter. The AVT filter used in our database was designed to remove noise within the band of interest and to provide a smoother and more regular source for feature extraction, which enhanced the pattern recognition capacities of the machine learning method. The original AVT algorithm was designed using sample discard; however, we modified the original algorithm to avoid sample discarding before the classifier, as presented in Figure 3.

**Figure 4:**
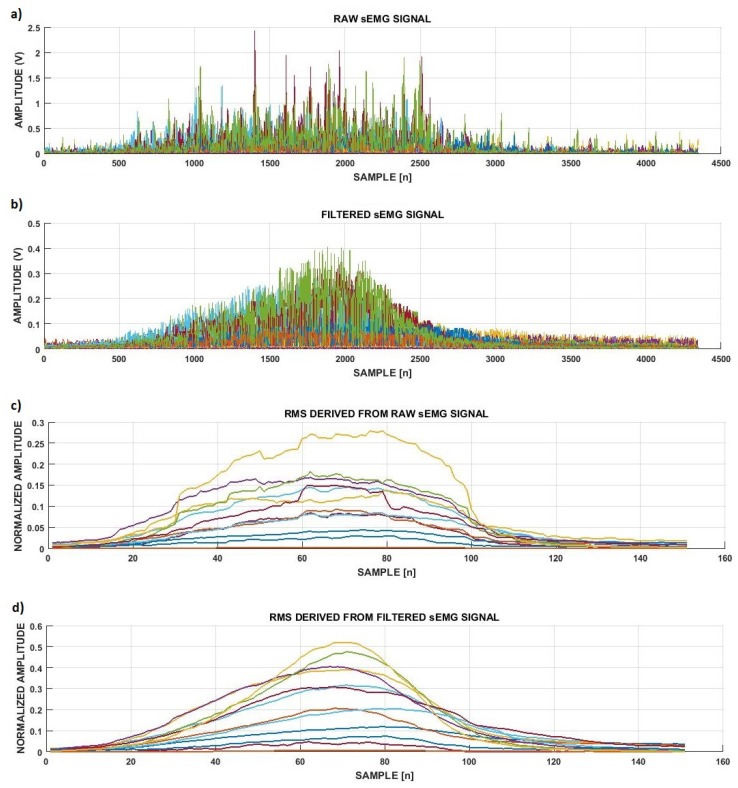
Effect of the AVT filter on the channels of the raw rectified sEMG signal and RMS feature for a movement repetition performed: (**a**) raw rectified sEMG signals; (**b**) the same portion of sEMG signals filtered; (**c**) RMS feature extracted from the signals presented in (**a**); (**d**) RMS feature extracted from the signals presented in (**b**).

The AVT filter, as designed, processed each segment (sg) of 200 ms extracted from the sEMG signal. Once a 200 ms segment was processed, the segmentation window slid 10 ms forward in the signal, characterizing a sliding-window approach. Considering the Mean Amplitude Value (MAV), the overall process itself was similar to a moving average filter; the main difference resides in the selective capacity provided by the *Mean Signal Deviation* (MSD), composed by the standard deviation of the signal (σ) rather than just the hard threshold based on the *Mean Signal Amplitude* (MSA) alone. After the definition of the range of excursion (MSA±MSD), the only samples (*s*) to be altered were those out of its boundaries, which were replaced by the MSA value, while the remaining samples stayed intact. The threshold also suffered from the influence of two pondering filter factors (ff1) and (ff2). The filter factors were used to provide a low-pass behavior for the 190-ms (95% of the signal) portion of the segment and highlight the dynamics of the 10-ms (5% of the signal) incoming portion for each sliding window. For that, the values of ff1 and ff2 were defined as 0.8 and 0.2, respectively. The effect of the designed AVT filter on the sEMG signal is presented in Figure 4a,b, as well as its effect on the RMS feature in Figure 4c,d. The Gaussian response derived from the signal was a direct consequence of the incremental activation profile of the Motor Units’ Action Potentials (MUAPs), which led to proportional EMG responses. More detailed information about the AVT filter and its comparison with other filtering and non-filtering scenarios was explored in [16].

### 2.5. Signal Segmentation and Feature Extraction

Generally, the definition of segment lengths and the selection of features is not a well-defined field in sEMG signal processing, with approaches varying considerably between those two factors. Despite the representativity of the sEMG signal generally being proportional to the segment length used [24], previous tests with the NINAPro database [5,12] observed that variations of 100 ms, 200 ms, and 400 ms of signal length did not offer a significant statistical difference for the results. Moreover, the use of more sophisticated frequency or time-frequency features also did not present a clear accuracy improvement compared to more simplistic signal representations for offline classification [12,25]. Furthermore, according to [26], there is no guarantee that additional individual efficient features of a model would offer a more efficient combination for signal classification considering that the systems tend to overfit. Previous studies [13,27] also concluded that due to the sEMG nature, a non-linear kernel is even more efficient for the signal classification than a specific set of features.

Although higher accuracy rates are obtained using bigger sEMG portions (windows), the process of signal buffering results in delays in the classification response that usually preclude the real-time control of assistive devices [24]. In our paper, the overlapped windows approach was chosen for the signal segmentation to maintain a balance between reasonable signal representation and the system responsiveness. Additionally, similar papers [12,13,14,15,28] were also considered to enable a fair comparison of results. Thus, overlapped windows of 200 ms of length and 10 ms of increment were used to segment the signal and extract the classical time-domain features: *RMS*, *Variance* (VAR), *Mean Absolute Value* (MAV), and *Standard Deviation* (SD), which are commonly used in sEMG signal classification [7,10,29], for each one of the 12 channels. This simple set of features for the signal representation was chosen based on previous related NINAPro works and also to highlight the consistency of our database. This approach also highlights the potential of our reliable classifiers, which can match or even outperform related results in the literature even when not using longer sEMG segments or more complicated signal representations.

### 2.6. Signal Classification

The signal classification was performed using state-of-the-art classifiers based on Extreme Learning Machines (ELM) in its standard (ELM) and Regularized (RELM) forms [30,31,32]. We derived a reliable version of the classifiers denoted R-ELM and R-RELM, respectively.

#### 2.6.1. ELM

The ELM is a particularly attractive machine learning solution for applications that demand a quick model formation. The classifier is formed through a non-iterative method that does not require the optimization process and instead uses a Moore–Penrose pseudoinverse that allows the achievement of an optimal model considering a tolerance for error. By its nature, the method natively avoids some classical problems of more traditional machine learning solutions such as local minimal and sub-optimal solutions [33]. Furthermore, the method has a natural multiclass capacity and a very reasonable computational cost when compared to the reference classifiers in the field, such as SVM [25]. The basic ELM structure is composed of the linear system presented in Equation (1), where *H* is the input matrix formed by the features projected by a kernel, β is the model to be found, and *T* is the label matrix. The derivation of *H* is detailed in Equation (2), where *w* and *b* are the random weight of the network neurons and bias, attributed within a range of [−1; 1] and [−1.5; 1.5], respectively, to maintain the low-pass response of the classifier. The ϕ represents the Radial Basis Function (RBF) kernel that projects each one of the *N* features in the *L* hidden neurons, a kernel acknowledged to be efficient for sEMG signal classification [13,27].

For the ELM, *L* is the only hyperparameter to be defined. Thus, we performed preliminary tests to relate the *L* number and accuracies in the training and testing of the models for both ELM and RELM classifiers within a range of 50–1000 hidden neurons. The optimal number of hidden neurons was defined by the maximum accuracy rate achieved for each subject.

(1) Hβ=T

(2) $$\;H=\left[\begin{array}{ccc} \phi (w_{1}x_{1}+b_{1}{,}_{1}) & \cdots & \phi (w_{L}x_{1}+b_{1}{,}_{L})\\ \vdots & \ddots & \vdots \\ \phi (w_{1}x_{N}+b_{N}{,}_{1}) & \cdots & \phi (w_{L}x_{N}+b_{N}{,}_{L}) \end{array}\right]$$

For the ELM and RELM, $H^{†}$ was calculated through the Moore–Penrose pseudoinverse and Tikhonov regularization (with *C* as the regularization factor), respectively, as presented in Equation (3). With $H^{†}$ defined, the system can be solved in a very straightforward manner, as presented in Equation (4) [33].

(3) $$H^{†}=H^{T}{\left(H^{T}H+\frac{I}{C}\right)}^{-1}T$$

(4) $$\beta =H^{†}T$$

**Figure 5:**
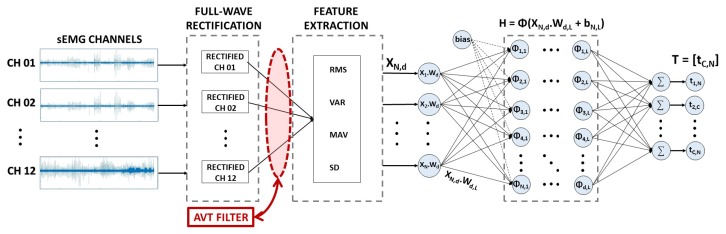
sEMG signal classification using a generic ELM classifier and the AVT filter. In this example, *N* is the number of samples to classify in *C* classes using *d* features and *L* hidden neurons.

The final label is attributed using an argmax heuristic where the highest output value (T) among all classes takes the label. Two metrics were used to appraise the system in its standard and reliable forms, the overall accuracy, presented in Equation (5), and the weighted accuracy, which ponders the accuracy for each one of the 18 classes (*c*), presented in Equation (6). The overall system architecture for the sEMG signal classification is presented in Figure 5.

(5) $$\mathrm{overall}\;\mathrm{accuracy}\;(\%)=\left(\frac{\mathrm{Correct}\;\mathrm{classifications}}{\mathrm{Total}\;\mathrm{samples}\;\mathrm{tested}}\right)\times 100\%$$

(6) $$\mathrm{weighted}\;\mathrm{accuracy}\;(\%)=\bar{\left(\frac{\mathrm{Correct}\;class_{c}\;\mathrm{classifications}}{\mathrm{Total}\;class_{c}\;\mathrm{classifications}}\right)}\times 100\%$$

#### 2.6.2. Reliable Signal Classification

**Figure 6:**
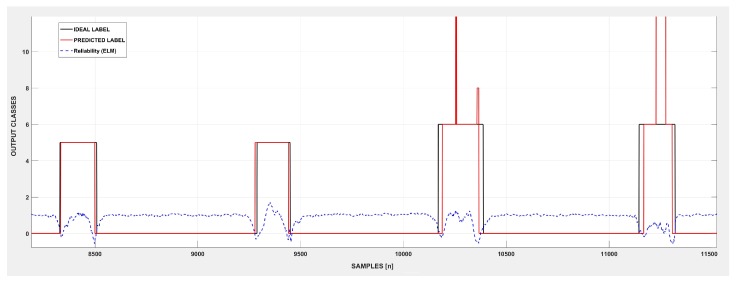
An example of the reliability metric in the ELM classifier: The solid black line provides the ideal label of the output class. The solid red line provides the predicted class, while the blue dashed curve represents the reliability of the system for each classification performed. Classification errors tend to occur when the reliability metric drops, which indicates the non-reliable classifications, which can be autonomously identified by the classifier.

To define the adequate class to label a determined input, the ELM method relies on the argmax heuristic. Once the model processes the input, the likelihood of belonging to each class is derived from the argmax value. Thus, the argmax output vector retains the probability of a particular sample belong to each class. The output class label for each sample is then attributed based on the higher argmax value, which in an ideal scenario is by far higher than the remaining classes, forming a reliable classification. Using this inherent mechanism of the ELM classifier to attribute labels, and based on the interval range calculated by Equation (7), a threshold(th) value was designed to identify the non-reliable classifications; instantly ignored by our classifier. Thus, the reliable version of the classifier can maintain a more coherent and robust classification and autonomously discard outliers and poorly-fitted data. An example of the variation of the maximum argmax value according to each classification performed is presented in Figure 6. The movement transitions are well known for the lack of signal representativity and class overlap in a machine learning perspective. Those factors make the class distinction in these sections particularly challenging, precluding an ideal class separation [34], and result in lower argmax values, which lead to lower reliability of the classification for those periods. The same situation occurs in the classification ripples in the intermediate portion of the signal that provoke an erroneous classification each time the classifier fails to reach an appropriate value of argmax and adequate class separation. The solid black line presents the ideal classification in Figure 6, while the solid red line represents the predicted class output. It is possible to note that the mismatches (errors) in the signal classification tended to occur for the drops of reliability in the classification, which is represented by the dashed blue line.

(7) $$th_{reliability}=\mu_{reliability}-\sigma_{reliability}$$

The value of threshold ($th_{reliability}$) was derived from the average ($\mu_{reliability}$) reliability (max(argmax)) of the signal, considering its standard deviation ($\sigma_{reliability}$). This factor provides a relaxation factor for the classifier given that some classifications performed with a slightly lower value than the $\mu_{reliability}$ are often correct. Our heuristic takes as the premise that if a representative dataset trained the classifier, a reliable test sample must provide patterns that are fitted enough in the trained model to offer consistent higher values of the argmax in comparison to remaining classes. In an ideal scenario, the correct class is related to an argmax output value, which is higher than the average, while the remaining classes achieve considerably lower value, characterizing an adequate class separation. This classification, when it occurs, characterizes a reliable classification. For non-satisfactory values of the argmax, we ignored the classification performed, since it is better not to perform any action than to provide an erroneous action that may harm the user or prejudice the environment that surrounds him/her. At the same time, this method enables criteria for data discarding and can be improved in the future to decide when the model needs to retrain and which classes are in fact capable of being learned by the classifier. The reliable versions of ELM and RELM classifiers are denoted in our paper as R-ELM and R-RELM, respectively. The regular value of reliability in Figure 6 is perceptible for the rest class and so is its sudden fall in movement transition sections of the classifications.

## 3. Results

### 3.1. IEE Database Validation

**Figure 7:**
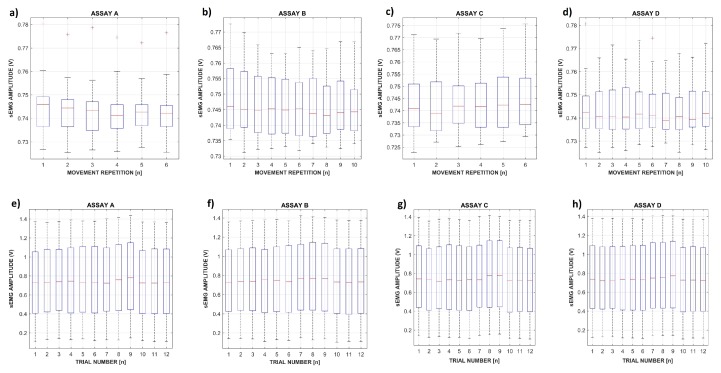
Distribution for sEMG amplitude after the rectification considering the four different assays: The evaluation considers all the movement repetitions and the 12 trials performed, with Trials 1–3, 4–6, 7–9, and 10–12 performed by Subjects 1, 2, 3, and 4, respectively.

Ideally, the data should be as representative and distinct as possible to enable the classifier’s optimal accuracy. Since several experimental factors such as electrode positioning, subjects’ physiology, and fatigue may alter the sEMG signal, we decided to evaluate the consistency of the IEE database. Figure 7 presents the average distribution of sEMG signals’ amplitude concerning movement repetitions and the 12 trials performed in each assay.

The first analysis showed that different assay types (A, B, C, and D) did not result in a significant difference regarding the average sEMG signal amplitude. However, the movement repetition itself was identified as an influencing factor that resulted in one outlier value. An ANOVA (p=0.05) was used to validate the rectified sEMG signal amplitude regularity using each assay separately. The results confirmed that the movement repetitions were executed differently by the subjects, which made them and ultimately the trials variable both significant factors. The signal dispersion concerning movement repetition was similar to E2 (Exercise 2) from the NINAPro database, as presented by Atzori et al. [13]. Regarding outliers, one of them was detected for each movement repetition of Assay A and Movement Repetitions 1 and 6 of Assay D. That was expected to occur in some assays as a consequence of the physiologic differences regarding the subjects, which was magnified by movement execution, generating distinct EMG activation profiles. Even so, Assays B and C did not present any outliers, considering the average signal amplitude.

**Figure 8:**
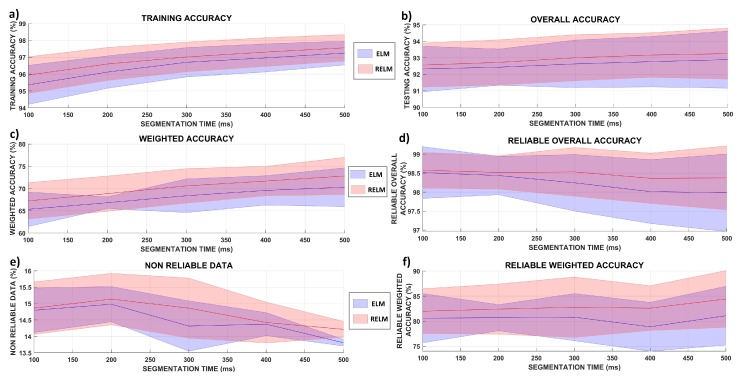
Influence of segmentation lengths in different metrics of the system: Notably, the standard versions of the classifiers tend to benefit slightly from more extensive lengths for feature extraction, while this does not affect the reliable form of the classifiers significantly.

Regarding the segmentation time, the related literature cites a trend of increasing the accuracy rate proportionally to the window length. We evaluated this aspect using the data from Assay A of Subject 1 of all repetitions to check the influence of segment window length on different metrics of the system, as presented in Figure 8. The evaluated metrics were: (1) training accuracy of the model (Figure 8a); (2) overall accuracy (Figure 8b); (3) weighted accuracy (Figure 8c); (4) reliable overall accuracy (Figure 8d); (5) non-reliable data rate (Figure 8e); and (6) reliable weighted accuracy (Figure 8f). Our test evaluated intervals of 100 ms, 200 ms, 300 ms, 400 ms, and 500 ms, for the same set of our original TDfeatures. The statistical significance for all tests performed was evaluated through an ANOVA (Tukey test, p=0.05). In this same test, the influence of ELM vs. RELM was also evaluated as a control variable.

For the training accuracy rate, it was found that the segmentation length had a statistical significance (which tended to be most influenced by the 400-ms and 500-ms scenarios), while the method of classification itself did not have a significant influence on the results. This suggests that longer windows for feature extraction are most likely to benefit the formation of a more accurate model. The same result was also verified regarding the non-reliable data detected by the classifier; the more extended segmentation tended to lead the system to narrower ranges at lower plateaus, which seems to reflect a situation of non-overfitting, which would increase the discarding of samples considerably. The overall and weighted accuracy metrics presented a significant response regarding the classification method, with RELM showing higher accuracy rates, but it was not significant for the segment size variation. The results suggest that despite longer segments influencing the training accuracy, this does not necessarily translate into higher accuracy rates. Thus, despite offering lower results, the 100-ms segmentation can be applied without any significant implications, at least following the test conditions. Both overall and weighted reliable metrics did not present statistically-significant differences in the variation of segment length or classification method. This result suggests that our reliable forms of the classifiers, at least for the conditions tested, were able to mitigate the influence of segmentation length in the accuracy results, proving to be robust alternatives to sEMG signal classification.

### 3.2. Signal Classification

Table 2 presents the classification results using ELM and RELM models in their standard and reliable forms (denoted R-ELM and R-RELM, respectively). The results exposed in Table 2 are organized by assays, subjects, methods, and two different metrics. The overall sample to sample results (overall accuracy) are given, formed by the comparison of the ideal and predicted labels, and the weighted accuracy, which considers the weighted average results among all classes. For the reliable versions of the classifiers, the average discard rate of the non-reliable data is also present. Each result considers the three repetitions performed by each subject in each type of assay (Trials 1–3 for Subject 1, 4–6 for Subject 2, 7–9 for Subject 3, and 10–12 for Subject 4).

A full-factorial design of experiments (p=0.05 and $R^{2}=85.4\%$) was conducted to define the factors that significantly affected the accuracy rate. The assay type, both classifiers (in their standard and reliable for ms), both accuracy metrics, and the subjects were considered controlled variables, while the accuracies were treated as the response to the experiment. All the variables excluding the classifiers’ individual interaction with assays and subjects and the mutual interaction with metric and assays and metric and subject were significant in the test. These results were coherent with the results presented in Table 2, which demonstrated distinct results and, therefore, the influence of all these factors on the results achieved.

The overall accuracy achieved rates above 90% in all cases and very close to 100% for several scenarios of the reliable versions of the classifiers. In contrast, the weighted accuracy, which considers all the movement classes to compose the final average, was lower for all tests. The use of both metrics was pertinent given that the overall accuracy is generally used in papers in the area, and the weighted accuracy presented accuracy without the bias caused by the rest class. There was a visible difference in the weighted accuracy among all the subjects, but a reasonable coherence in the rates considering the sequential (A and B) and random (C and D) assays performed in the baseline and the reliable form of the classifiers. The number of repetitions used proved to be insignificant concerning the use of four (Assays A and C) or six (Assays B and D) movement instances to train the classifier.

### 3.3. NINAPro Databases’ Classification

To validate and compare our classifiers with related papers, we performed the classification of the whole of Databases 1, 2, and 6 (DB1, DB2, and DB6) of the NINAPro database formed by its three different “exercises” (E1, E2, and E3) comprehending 50 different upper-limb movements (DB1, DB2) and 18 hand and force movements with assays’ repetition (DB6). The average results achieved for each database are presented in Table 3. For the sake of comparison, we chose not to detail each database result, but to use an average of the result concerning the exercises E1, E2, and E3, as presented in the related papers. The results derived from DB6 were tested for two different conditions. The first Condition (CD1) consisted of the intra-session signal classification, while the second Condition (CD2) used data from different assays to train and test the classifier, as presented by Palermo et al. [15].

As Palermo et al. [15], the intra-session results were far superior to the cross-session signal classification. However, our results presented higher accuracy than those of the related paper, which used only the mean amplitude value and wavelength as input features, as presented in Table 3.

The DB1 database gathered the same 50 distinct movements of DB2 with differences in data acquisition. The average accuracies presented in Table 3 was calculated based on the related papers’ best scenario comprehending the 27 subjects of the database who performed ten repetitions of each movement. Our baseline classifiers were slightly less accurate in this database; however, the reliable form of the regularized ELM was able to match the state-of-the-art rates.

Regarding the NINAPro DB2, the results presented in Table 3 were the best case scenarios of the baseline classifiers described in the related papers composed by the average results derived from 40 subjects. Although the baseline forms of our classifiers were slightly less accurate than the accuracies of the related papers, the reliable versions of our classifiers were capable of outperforming these rates. Moreover, the length of the windows used in the segmentation process is a factor to be considered, since generally, accuracy tends to increase proportionally to the length of the sEMG signal used in the signal classification.

All the comparative tests were conducted using the same movement samples (i.e., Movement Repetitions 1, 3, 4, and 6 to train and Movement Repetitions 2 and 5 to test in DB2) and the data ratio to train and test (i.e., 50% for training and 50% for testing in DB1 and DB3) of the related papers. However, differently from the related papers, the features used were those indicated in Section 2.5 of our paper.

## 4. Discussion

Regarding the IEE database, it was perceptible that the order of execution of movements had a direct impact on the accuracy achieved. The sequential order assays (A and B) had significantly higher accuracy than those formed by random movements (C and D). In our perspective, this is caused probably by the subjects’ learning capacity, implying more precise and regular movement execution concerning timestamps and the emphasis of the movement itself. In random assays, the movements can sometimes confuse the subject, generating an error factor, which generally appears magnified in Assay D, which had double the random repetitions and the lowest plateau of accuracy rates among all assays. The sequential order Assays A and B had consistent average results with a difference of ≈2% in weighted accuracy and less than 1% in the overall accuracy, which demonstrates the regularity of the classifiers in the identification of signals derived from different movement repetitions (two repetitions tested in Assay A and four repetitions tested in Assay B). These results may indicate that four representative movement repetitions were enough to train a reliable model to be tested with future *n* instances and still maintain the classification consistency (not considering some experimental precluding factors such as noise, electrode displacement, etc.).

A significant difference between the accuracies achieved by different subjects was also perceptible. Even using the same experimental protocol and electrode positioning, different subjects tended to present distinct bioelectric characteristics that influenced the signal classification. According to Atzori et al. [14], younger and skinnier subjects tended to achieve higher accuracy rates. Assuming the assessment of Atzori et al. [14] is true, the higher accuracy observed for Subject 2 was potentially linked to his thinner fat tissue, which may have helped in acquiring more representative sEMG signals. Subjects with thicker fat tissue usually tended to attenuate the signal amplitude, tending to preclude the optimal signal pattern recognition. Subject 2 also presented a lesser standard deviation related to the weighted accuracy and generally had among the lesser discard rates concerning non-reliable data. The worst results were achieved by Subject 4 who had a higher Body Mass Index (BMI) among the population involved in the study. An exception occurred in Assay C, where Subject 1 reached a lower accuracy due to the outlier Repetition 2 that reached an 11% accuracy rate in the weighted accuracy metric. This value is highly unusual and should be treated as an outlier.

The number of hidden neurons is an essential factor to define from the perspective of machine learning. Since this is not the main point of our paper, we decided to define the number based on the number that provided us with a higher accuracy rate. However, there are more sophisticated approaches in information theory generally based on the Bayesian or Akaike information criterion and pruning algorithms that aim to balance the amount of useful information used in the creation of the model and the computational weighted accepted as function of a proposed application. The regularized form of the baseline ELM classifier tended to achieve slightly higher results (usually ≈2%) due to its capacity to be more resilient to input pattern variations. For all assays and trials, the reliable forms of the classifiers were able to boost the classification accuracy in all scenarios by eliminating the non-reliable classifications. The outcome of this sample discard was observable in both accuracy metrics, but the practical effect was more visible in the weighted accuracy where the best results reached improvements close to 20%. The more outstanding improvement rate achieved occurred for Subject 4 on Assay D with more than a 23% accuracy improvement. The data discard was consistent in every assay, varying between ≈7.8% and ≈14.4% (both on Assay C). The R-RELM discarded more samples in every scenario due to the more regular value of its argmax value, a consequence of a regularized method, less sensitive to outlier sample inputs.

**Figure 9:**
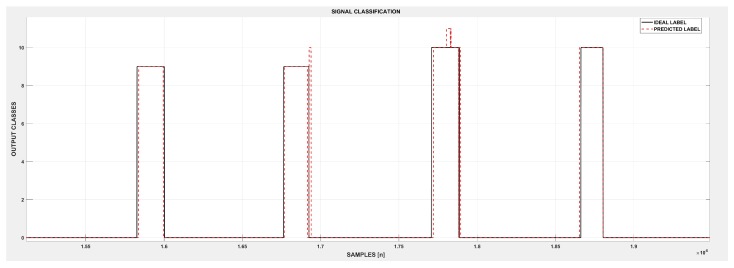
A label comparison plot: Through the analysis of the ideal and predicted label, it is possible to estimate the classification errors and propose specific solutions. In the figure, two repetitions of Movements 9 and 10 performed by Subject 1 in Assay A were used to illustrate the capability of the method.

Regarding both metrics used, the weighted accuracy presented a more balanced alternative to evaluate the system considering that the sEMG databases are frequently unbalanced datasets, which tend to have more samples from the rest class than actual movements, causing a bias in the overall accuracy rate. Given its average lower amplitude, the rest class is the most reliable “movement” to classify [13], reaching values close to 99%. However, the overall accuracy (sample to sample classification) still provided valuable information regarding time, enabling a more precise evaluation of error occurrence in classification. Thus, it is possible to work around solutions to prevent and correct these errors, which are frequently related to the signal representativity, as presented in Figure 9, which compares the desired label (solid black line) with the predicted label (dashed red line). Despite the excellent signal prediction performed for the two sequential movements repetitions (Classes 9 and 10), the classification ripples at the end of the second repetition of Movement 9 and the middle portion of Movement 10 were present. Both ripples shifted the predicted label to Classes 10 and 11, respectively. The initial portion of the first repetition of both movements also contained a slight delay for the movement classification and in the second repetition of Movement 10. The predicted label was advanced in time compared to the ideal label. Classification errors in the signal transition are well known in the literature, although, upon an offline analysis, these errors could be in part attributed to the non-ideal relabeling process. The sample to sample plot was especially useful to identify specific classification drawbacks such as misclassification ripples. The correspondent accuracies achieved for Movements 9 and 10 using the weighted accuracy metric were 85.82% and 90.78%, respectively. Thus, although the weighted accuracy is a fairer comparison, the overall accuracy used along label comparison plots can still provide valuable information.

In comparison with related work, Atzori et al. [14] and Kuzborskij et al. [12] related average accuracy rates of 76% and 75%, respectively, in their best case scenario using Database 1 (DB1) of NINAPro. DB1 is composed of the same 52 classes of DB2 with differences regarding the experimental protocol in the signal acquisition and number of movement repetitions. To enable a fair comparison, we used the same movement repetitions to train and test our classifiers. However, two basics differences were the input features of the related work that also contemplated features in the frequency domain and the segment length for feature extraction. Despite our approach dealing only with features in the time domain and using segments half as long as the related papers, we were still capable of matching the best results related to our R-RELM method that, among others, contain the same 17 movements performed in our database. The segment length is an important factor to consider since longer signal portions tend to lead the accuracy to higher rates at the cost of system responsiveness [24]. Instead of using a sliding-window of 400 ms, we preferred to use segments of 200 ms and to leave a margin to improve in this context. The features in the frequency domain are commonly used to detach the signal representation from the amplitude-based metrics exclusively and tend to improve the accuracy rates when used in combination with time-domain features. Despite test several features in both domains, Kuzborskij et al. [12] found their best results using two time-domain features. Thus, we decided to use more straightforward features and avoid others that may overload the signal processing, which was enough to match both related papers with a difference of ≈1% from Atzori et al. [14], who used six different features in both domains.

Regarding the papers that used the NINAPro DB2 database, as presented in Table 3, our classifiers were slightly less accurate, but compatible in their baseline forms and outperformed the referenced papers with the reliable forms of our classifiers. Once more, our classifiers were able to reach these results even using a signal segment half as long as the other papers and avoiding the use of frequency domain features, common to all of them. The paper of Zhai et al. [36] had the closest accuracy compared to our method. Zhai et al. used a deep learning classifier based on a Convolutional Neural Network (CNN) and an adaptive method to classify the NINAPro DB2; in their best results considering the baseline methods, they reached an average of 78.7%. As typical of CNN-based methods, their method relied on a considerable amount of processing to generate the models, which in their best case scenario reached an average accuracy close to 80% using their adaptive method. The reliable versions of the classifiers were able to achieve an average accuracy close to 80.0% for the NINAPro DB2 using our feed-forward method, without any retraining, keeping a more straightforward approach, but as accurate as the adaptive CNN.

The paper of Palermo et al. explored the recently-created NINAPro DB6, which is composed of signals acquired on five different days and made with the upper-limb movements focused on the gripping of different objects. Based on Kuzborskij et al. [12], the authors used the mean amplitude value and the wavelength of the signal as input features to test the accuracy of the method to classify signals from the same trial and in cross-sections of the database. On their best-case scenario by using the combination of both features, they reached an average accuracy of 52.43% for the data derived from the same trial of each user (CD1) and 25.40% when using cross-section data (CD2), mixing the data from different assays. This decrease in accuracy rate is expected since even for the same person, the characteristics of the sEMG signal tend to change in time, changing the signal morphology and, as a consequence, the features extracted. Our method was able to reach higher accuracies in both cases for all classifiers using the same length for the segmentation window and our four features in the time-domain. The reliable versions were capable of enhancing by ≈16% the accuracy rate in both scenarios.

The IEE database presented consistent results with both metrics and in the best case scenario of the R-RELM method achieved average results of 85.41% for accuracy rate considering all the trials and assays performed and the weighted accuracy metric. For each independent assay, the results of weighted accuracy were 90.00%, 86.95%, 80.21%, and 78.14% for Assays A, B, C, and D, respectively. For the overall accuracy metric, the same tests reached average accuracies of 99.05%, 99.21%, 98.68%, and 98.89% for the same scenarios. The higher accuracy rate in favor of the sequential and smaller assays tended to indicate the influence of the subjects on the classification process. For the sequential order assays, subjects tended to execute the movements more similarly, while random assays led to subjects to execute the movements in a more improvised way. Those small differences in the movement execution tended to affect the sEMG signal morphology and consequently its classification. Considering the possible variations of the movement execution (and the muscular activation related) and the number of repetitions, despite the initial 66% of data being able to be used successfully to train the classifiers for adjacent samples, further studies yet must approach more detailed problems derived from prolonged usage. Overall, the IEE database presented comparable rates or higher with the NINAPro database, despite the fact that it only contained movements from Exercise 2 of the NINAPro database.

## 5. Conclusions

We evaluated the IEE sEMG database, which we are making available to download on our website (www.ufrgs.br/ieelab). Additionally, we presented the reliable versions of two ELM-based classifiers and the effects of data discard and accuracy rates reached by the regularized and the standard versions of each classifier. The reliable classification appeared to mitigate even the earlier stages of processing such as the segmentation times for feature extraction. We also presented a stochastic and practical filtering method to achieve smoother features from the signal, improving its representativity to achieve reasonable accuracy rates that may in the future help with using those tested algorithms in a real-time prosthetic application. All the results were evaluated by two metrics: the overall accuracy and the weighted accuracy. We also evaluated our pre-processing strategies and classifiers using three different databases from NINAPro.

The accuracy results achieved provided the experimental validation of the gathered data and the reliable forms of the ELM classifiers. We hope the IEE database can help the scientific community by providing a benchmark database, as well as all the supplementary materials related such as codes/routines, videos, and procedures, which will hopefully support the development of natural prosthetic control methods and the general development of this research field. While using our database, we strongly encourage all the users to use both metrics of evaluation since the rest class could bias the overall accuracy result. However, the use of label comparison plots in overall accuracy is also useful to check where classification errors occur and to propose specific solutions. The reliable forms of our ELM classifiers (especially in its regularized from) were shown to match or even outperform some state-of-the-art methods using a very straightforward approach. Since we were able to identify the reliable samples autonomously for the classification, further developments must focus on the development of regenerative classifiers. In this regenerative classification, the classifier must be capable of identifying a class with poor fitting and autonomously request for a more updated sample to refresh the classification model. We believe that it must be capable of maintaining stable accuracies for long-term classification and also help to enhance the cross-session/multiuser problem of limited accuracy.

## Figures and Tables

**Table 1:** IEE database subject description (F.C: Forearm Circumference, F.L: Forearm Length (F.L)).

SUBJECT	LATERALITY	GENDER	AGE	HEIGHT (m)	WEIGHT (kg)	F. C. (cm)	F. L. (cm)
**1**	Right-Handed	Male	31	1.87	78.0	28.3	25.3
**2**	Right-Handed	Male	26	1.80	70.0	27.0	24.0
**3**	Right-Handed	Female	29	1.64	60	25.4	22.7
**4**	Right-Handed	Male	34	1.82	84.0	27.8	25.7

**Table 2:** Mean accuracy rates achieved considering the three repetitions for each assay. The results are divided by assay, subject, method, and accuracy. For the reliable versions of the classifiers, the amount of discarded data is also present. R-RELM, Reliable Regularized ELM.

ASSAY A					ASSAY B				
**SUBJECT**	**METHOD**	**WEIGHTED** **ACCURACY (%)**	**OVERALL** **ACCURACY (%)**	**NON-RELIABLE** **DATA (%)**	**SUBJECT**	**METHOD**	**CLASS** **ACCURACY (%)**	**OVERALL** **ACCURACY (%)**	**NON-RELIABLE** **DATA (%)**
**1**	**ELM**	72.17 ± 10.80	94.00 ± 1.13	-	**1**	**ELM**	71.24 ± 12.40	94.22 ± 1.48	-
	**RELM**	74.60 ± 10.82	94.51 ± 1.10	-		**RELM**	72.56 ± 13.07	94.51 ± 1.39	-
	**R-ELM**	85.50 ± 16.37	98.80 ± 0.33	13.22 ± 1.76		**R-ELM**	82.63 ± 13.16	98.45 ± 0.60	11.34 ± 0.43
	**R-RELM**	89.25 ± 15.38	99.16 ± 0.26	13.15 ± 1.61		**R-RELM**	85.43 ± 14.30	99.06 ± 0.40	12.27 ± 0.78
**2**	**ELM**	80.16 ± 8.70	95.73 ± 2.20	-	**2**	**ELM**	82.27 ± 7.62	96.48 ± 0.56	-
	**RELM**	80.98 ± 8.11	95.86 ± 2.10	-		**RELM**	83.09 ± 7.07	96.67 ± 0.46	-
	**R-ELM**	91.06 ± 7.82	98.87 ± 0.93	11.03 ± 1.58		**R-ELM**	92.61 ± 5.52	99.18 ± 0.35	11.10 ± 0.66
	**R-RELM**	92.57 ± 6.70	99.17 ± 0.83	11.67 ± 1.21		**R-RELM**	96.00 ± 3.22	99.60 ± 0.29	11.72 ± 0.51
**3**	**ELM**	72.20 ± 11.37	93.65 ± 1.15	-	**3**	**ELM**	72.54 ± 11.62	93.74 ± 0.47	-
	**RELM**	74.17 ± 11.10	94.10 ± 1.02	-		**RELM**	74.55 ± 10.86	94.17 ± 0.41	-
	**R-ELM**	86.56 ± 10.26	98.76 ± 0.61	13.53 ± 0.20		**R-ELM**	84.82 ± 15.47	98.57 ± 0.26	12.98 ± 0.88
	**R-RELM**	91.40 ± 7.29	99.17 ± 0.40	13.93 ± 0.47		**R-RELM**	88.04 ± 14.45	99.00 ± 0.13	13.28 ± 0.81
**4**	**ELM**	64.60 ± 12.10	93.75 ± 0.57	-	**4**	**ELM**	62.65 ± 13.12	93.79 ± 0.26	-
	**RELM**	67.34 ± 12.56	94.23 ± 0.47	-		**RELM**	63.67 ± 12.98	93.98 ± 0.40	-
	**R-ELM**	83.33 ± 13.26	98.53 ± 0.46	13.01 ± 0.36		**R-ELM**	74.89 ± 12.82	97.89 ± 1.43	10.57 ± 3.81
	**R-RELM**	88.47 ± 11.57	99.02 ± 0.46	13.31 ± 0.35		**R-RELM**	82.83 ± 10.21	99.16 ± 0.13	13.28 ± 0.43
**1**	**ELM**	42.23 ± 18.31	89.25 ± 5.07	-	**1**	**ELM**	60.73 ± 16.30	92.97 ± 0.39	-
	**RELM**	43.24 ± 17.91	89.43 ± 5.62	-		**RELM**	62.12 ± 16.18	93.25 ± 0.60	-
	**R-ELM**	47.33 ± 23.57	97.62 ± 1.10	13.30 ± 1.76		**R-ELM**	73.26 ± 24.76	98.46 ± 0.38	14.02 ± 3.14
	**R-RELM**	50.48 ± 23.40	97.64 ± 1.63	13.23 ± 1.50		**R-RELM**	77.31 ± 21.86	98.80 ± 0.35	13.53 ± 2.43
**2**	**ELM**	73.81 ± 10.34	94.42 ± 20.90	-	**2**	**ELM**	70.05 ± 12.22	94.00 ± 2.13	-
	**RELM**	74.89 ± 10.22	94.70 ± 0.98	-		**RELM**	71.28 ± 11.95	94.27 ± 2.04	-
	**R-ELM**	84.21 ± 9.88	97.31 ± 2.47	7.76 ± 6.73		**R-ELM**	83.84 ± 11.09	97.83 ± 2.05	10.28 ± 1.95
	**R-RELM**	93.38 ± 6.27	99.28 ± 0.46	12.23 ± 0.28		**R-RELM**	87.92 ± 11.25	98.90 ± 0.95	12.51 ± 0.85
**3**	**ELM**	63.59 ± 13.20	93.12 ± 0.43	-	**3**	**ELM**	58.98 ± 14.60	92.67 ± 1.12	-
	**RELM**	65.49 ± 12.40	93.50 ± 0.68	-		**RELM**	60.85 ± 14.22	93.00 ± 1.17	-
	**R-ELM**	78.73 ± 14.00	98.00 ± 0.19	11.30 ± 1.02		**R-ELM**	74.50 ± 15.52	98.36 ± 0.68	11.38 ± 0.54
	**R-RELM**	80.22 ± 16.68	98.59 ± 0.22	12.11 ± 0.74		**R-RELM**	79.00 ± 17.25	98.97 ± 0.25	12.03 ± 0.68
**4**	**ELM**	49.17 ± 16.70	91.05 ± 0.95	-	**4**	**ELM**	50.06 ± 15.20	91.50 ± 0.56	-
	**RELM**	51.97 ± 17.11	91.56 ± 1.27	-		**RELM**	50.70 ± 15.66	91.59 ± 0.67	-
	**R-ELM**	66.65 ± 15.34	97.96 ± 0.26	13.90 ± 1.93		**R-ELM**	71.34 ± 12.41	98.12 ± 0.98	12.57 ± 0.40
	**R-RELM**	77.14 ± 17.34	98.54 ± 0.67	14.40 ± 1.10		**R-RELM**	74.38 ± 14.98	98.65 ± 0.43	13.33 ± 0.69

**Table 3:** Results and comparison with related papers in the area considering the three different NINAPro databases. CD, Condition.

PAPER	SEGMENT	DATABASE		AVERAGE ACCURACY (%)			
Kuzborskij et al. [12]	400 ms + 10 ms	DB1		75.00			
Zhai et al. [35]	200 ms + 100 ms	DB2		77.41			
Gijsberts et al. [27]	400 ms + 10 ms	DB2		77.48			
Atzori et al. [13]	200 ms	DB2		75.27			
Atzori et al. [14]	400 ms + 10 ms	DB1		76.00			
Zhai et al. [36]	256/184 points (Hamming window)	DB2		78.71			
Palermo et al. [15]	200 ms + 10 ms	DB6	CD1	52.43			
			CD2	25.40			
				ELM	RELM	R-ELM	R-RELM
This work	200 ms + 10 ms	DB1		68.77	71.63	73.13	75.03
		DB2		73.67	74.43	79.33	79.77
		DB6	CD1	64.72	65.21	68.43	69.83
			CD2	37.74	38.93	39.91	41.75
